# Decreased angiogenesis as a possible pathomechanism in cervical degenerative myelopathy

**DOI:** 10.1038/s41598-021-81766-8

**Published:** 2021-01-28

**Authors:** Christian Blume, M. F. Geiger, M. Müller, H. Clusmann, V. Mainz, J. Kalder, L. O. Brandenburg, C. A. Mueller

**Affiliations:** 1 Department of Neurosurgery, RWTH Aachen University, Pauwelstrasse 30, 52074 Aachen, Germany; 2 Institute of Anatomy, Rostock University Medical Center, Gertrudenstrasse 9, 18057 Rostock, Germany; 3 Department of Neuroradiology, RWTH Aachen University, Pauwelstrasse 30, 52074 Aachen, Germany; 4 Department of Medical Psychology and Medical Sociology, RWTH Aachen University, Pauwelsstrasse 19, 52074 Aachen, Germany; 5 Department of Vascular Surgery, Gießen University, Rudolf-Buchheim-str. 7, 35392 Gießen, Germany

**Keywords:** Neurological disorders, Neurological disorders, Biomarkers, Medical research, Immunopathogenesis, Inflammation

## Abstract

Endogenous immune mediated reactions of inflammation and angiogenesis are components of the spinal cord injury in patients with degenerative cervical myelopathy (DCM). The aim of this study was to identify alteration of certain mediators participating in angiogenetic and inflammatory reactions in patients with DCM. A consecutive series of 42 patients with DCM and indication for surgical decompression were enrolled for the study. 28 DCM patients were included, as CSF samples were taken preoperatively. We enrolled 42 patients requiring surgery for a thoracic abdominal aortic aneurysm (TAAA) as neurologically healthy controls. In 38 TAAA patients, CSF samples were taken prior to surgery and thus included. We evaluated the neurological status of patients and controls prior to surgery including NDI and mJOA. Protein-concentrations of factors with a crucial role in inflammation and angiogenesis were measured in CSF via ELISA testing (pg/ml): Angiopoietin 2, VEGF-A and C, RANTES, IL 1 beta and IL 8. Additionally, evaluated the status of the blood-spinal cord barrier (BSCB) by Reibers´diagnostic in all participants. Groups evidently differed in their neurological status (mJOA: DCM 10.1 ± 3.3, TAAA 17.3 ± 1.2, *p* < .001; NDI: DCM 47.4 ± 19.7, TAAA 5.3 ± 8.6, *p* < .001). There were no particular differences in age and gender distribution. However, we detected statistically significant differences in concentrations of mediators between the groups: Angiopoietin 2 (DCM 267.1.4 ± 81.9, TAAA 408.6 ± 177.1, *p* < .001) and VEGF C (DCM 152.2 ± 96.1, TAAA 222.4 ± 140.3, *p* = .04). DCM patients presented a mild to moderate BSCB disruption, controls had no signs of impairment. In patients with DCM, we measured decreased concentrations of angiogenic mediators. These results correspond to findings of immune mediated secondary harm in acute spinal cord injury. Reduced angiogenic activity could be a relevant part of the pathogenesis of DCM and secondary harm to the spinal cord.

## Introduction

Continuous compression and repetitive micro-traumas to the spinal cord define degenerative cervical myelopathy (DCM) as a unique entity^1^. Apart from chronic, mechanical harm to the spinal cord, research on the disease has focused on secondary pathophysiological pathways of endogenous immune-mediated reactions^2–6^. In particular, inflammation and angiogenesis seem to have a relevant influence on ongoing pathophysiological reactions in this chronic disease^2,5–7^. Inflammatory cells are detectable in the early and late onset of DCM in the spinal cord^8^. Activated macrophages/microglia are the predominant cell type and mainly responsible for inflammatory reactions in DCM^8^. Migration of these cells and activation of inflammatory reactions in the central nervous system (CNS) is mediated by fractalkine (CX3CL1)^9^. Regarding these pathomechanisms, inflammatory immune mediated reactions seem to be an important part of the pathophysiology of DCM. It was shown, that numerous cytokines trigger neuroinflammation in different entities. Previous research has suggested a contribution of IL-1 in acute CNS diseases such as SCI, cerebral ischemia, trauma, and subarachnoid hemorrhage^10–16^. Furthermore, neurodegeneration and neuronal death in neurodegenerative diseases are primarily due to increased levels of proinflammatory and neurotoxic mediators, amongst others IL-8 and CCL5 (Rantes)^17,18^.

The pathomechanisms of angiogenesis have been described in the context of DCM and should be taken into account as mechanisms of secondary injury to the spinal cord as well^2,5–7^. Additionally, it was demonstrated that pathological alterations in DCM are similar to spinal cord ischemia^19,20^. Ischemia deteriorates perfusion of the oligodendrocytes, promotes cellular death via apoptosis and demyelination^21^. Other pathophysiological factors of DCM include disturbed intracellular energy metabolism and mediated cell injury^22^.

In this context, Vascular Endothelial Growth Factor (VEGF) is a well-known factor of vascular angiogenesis, homeostasis and pathology^23^. In ischemia, injury and cerebral degeneration, multiple factors of the VEGF family are responsible for pathologies like atherosclerosis, blood vessel disease, BBB leakage, cerebral edema and neurogenesis during CNS repair and remodeling^24^. VEGF is associated with the cysteine knot growth factor family^25–27^. Furthermore, VEGF family includes VEGF A, -B, -C, -D and placental growth factor (PlGF)^28^. Three individual receptors (VEGFR 1, -2, -3), which apply to the tyrosine kinase receptor family. VEGFR-2 (kinase domain-containing receptor in humans) is an important mediator of angiogenesis via binding VEGF A, VEGF C and VEGF D^29,30^. VEGF signals are predominantly proangiogenic, although some interactions are more complex. VEGFR 1 downregulates angiogenesis by preventing VEGFR-2 from binding VEGF A^31–33^. In the context of angiogenesis, VEGF A is a good example for biphasic capacity in reestablishing neurovascular components. On the one hand, VEGF A promotes angiogenesis by stimulating the proliferation and migration of endothelial cells under pathological conditions. On the other hand, VEGF also has destructive characteristics, possibly leading to BBB leakage and brain edema after injury^34^. One of the VEGF C functions is the regulation of lymph-angiogenesis^35–37^. The lymphangiogenic capability of VEGF C is mediated by a VEGFR 3 receptor^38,39^. VEGF C–deficient animal models detected defects of lymphatic vessels^40^.

Another neuroprotective agent is Angiopoietin (Ang). Ang, and its receptor Tie, are endothelial growth factors. Ang was identified as a tissue-specific receptor in the Tyr kinase system. Members of the Ang family are Ang 1, Ang 2, Ang 3, and Ang 4^41,42^. Concerning the regulation of blood vessels, Ang 1 and Ang 2 have concurrent properties, via binding to Tie2 receptors^41^. Ang 2 is released under specific conditions from Weibel–Palade bodies of endothelial cells. Furthermore, Ang 2 operates as a receptor antagonist in the presence of Ang 1, but has agonistic attributes in the absence of Ang 1^43–45^. Apart from angiogenesis, Ang 2 plays a significant role in adult neurogenesis^46,47^. Léna et al. described that Ang 2 promotes the proliferation and migration of adult neuronal precursor cells (NPCs)^47–49^. Administration of Ang 2 stimulates the proliferation of NPCs in the CNS and promotes cell migration during neuronal development^49^.

Although aSCI is a different entity, there a several fundamental characteristics which correspond to DCM. In particular, the trauma to the spinal cord is the most important similarity between these two diseases. Concerning endogenous mediators (inflammation/angiogenesis), different alterations of concentrations have been reported in the context of aSCI^50–54^. Interestingly, inflammatory factors predominantly present with increased activation in the secondary cascades of endogenous reactions, whereas angiogenic mediator-activity decreases in SCI^50–52,55^.

Chronic compression of the spinal cord leads to decreased capillary density in gray and white matter^2^. Additionally, the numbers of endothelial cells, which play an important role in the integrity of the blood-spinal cord barrier (BSCB) are decreased as well^2,5^. BSCB disruption seems to be a crucial pathomechanism in the disease of DCM and angiogenic reactions initiate the majority of the pathological cascades of BSCB^2,50,56,57^. Thus, apart from mechanical harm to the spinal cord, inflammation and angiogenesis seem to be significantly associated with DCM.

In this study we aimed to detect alterations of inflammatory and angiogenetic mediators as sign of secondary pathomechanisms in DCM patients.

## Material and methods

### Study procedure and subject characteristics

**Figure 1:**
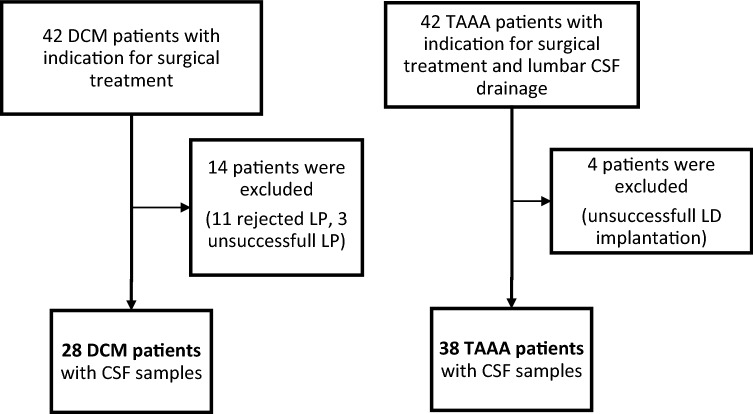
Flowchart of enrolled patients (DCM) and controls (TAAA) and finally included subjects. *CSF* cerebrospinal fluid, *DCM* degenerative cervical myelopathy, *LD* lumbar drainage, *LP* lumbar puncture, *TAAA* thoracic-abdominal aortic aneurysm.

The study was approved by the local ethics committee of the Medical Faculty of the Aachen University, RWTH (EK 164/13). All participants of this study gave written informed consent in accordance with the Declaration of Helsinki (Medical Association 2008). A total of 42 DCM patients and 42 patients with thoracic aortic abdominal aneurysm (TAAA) were prospectively enrolled in this study (Fig. 1).

Patients with DCM and indication for cervical decompressive surgery were potential subjects to be included. DCM patients with neurological deficits, not primarily caused by DCM or neurodegenerative disorders [e.g. amyotrophic lateral sclerosis, multiple sclerosis (MS); history of cerebral stroke, infections of the central nervous system (CNS) or spinal trauma] were excluded from the study. Patients of the DCM group underwent lumbar puncture (LP) preoperatively. 11 patients declined LP, in 3 patients LP was unsuccessful. These patients were excluded from the study.

Patients with TAAA and indication for surgical intervention formed the control group. TAAA patients routinely received preoperative lumbar drainage (LD) for intrathecal pressure monitoring during surgical procedures. Presence of any kind of neurological deficits or history of neurological disease were an exclusion criteria for TAAA controls. Implantation failed in 4 cases. These controls were excluded from the study (Fig. 1).

Overall, CSF samples were obtained in 28 patients of the DCM group and in 38 patients of the TAAA group. Patients and controls with CSF samples were included in the study for further investigation. (Fig. 1). We only took measurements without any missing values and mediators into account. Thus, sample sizes differed between routine CSF values, inflammatory and angiogenic mediators, as described in the analysis data section.

The neurological status of both groups was evaluated by clinical examination, modified Japanese Orthopedic Association Score (mJOA, Benzel et al.) (normal function: 18 points; mild myelopathy: 15–17 points; moderate myelopathy: 12–14 points; severe myelopathy: 0–11 points) and Neck Disability Index (NDI)^58–60^. The NDI provides information about limitations in patients’ everyday life caused by neck pain. Patients with higher values of NDI have more severe limitations.

### Samples and analysis

CSF and blood serum samples were taken prior to surgery from patients and controls. For further examination of inflammatory and angiogenic factors the samples were immediately deep-frozen at − 80 °C. The following routine laboratory findings of CSF were assessed: white blood cell count (/µl), lactate (mmol/l), protein concentration (g/l) and Glucose (mg/dl).

### Reibers’ diagnostic/blood-spinal cord barrier

To ensure that the measurements of endogenous mediators were limited to the intrathecal space and were not influenced by other systemic reactions, Reibers’ Diagnostic was performed from each sample (CSF/blood serum) of patients and participants of the control group. In doing so, the condition of the BSCB was assessed. Samples were examined via simultaneous nephelometric quantification (BN ProSpec System, Siemens Helathineers) for Albumin. Quotients for Albumin (AlbuminQ n × 10–3) and individual age-related references (AlbuminQ = (4 + age/15) × 10–3) were calculated^61,62^.

### ELISA testing

To evaluate alterations of crucial angiogenetic and inflammatory mediators, CSF samples of DCM patients and TAAA controls were examined via ELISA. The total levels of secreted factors in the CSF were quantified by Luminex xMAP technology in duplicates on a Luminex 200 system or by ELISA. VEGF A, interleukin-1β (IL 1 beta) and IL-8 were detected using the Procarta Multiplex (ThermoFisher, Dreieich, Germany; PPX-03). VEGF C or Rantes were detected using Singleplex for Luminex (Merck Millipore, Darmstadt, Germany; HAGP1MAG-12K-01). Ang 2 was determined using ELISA kit (ThermoFisher; KHC1641). The assays were conducted as recommended by the manufacturer.

### Data analysis

All data analyses were performed using IBM SPSS Statistics Version 25 (IBM Corporation, Armonk, NY). The groups’ CSF routine values were compared using a Multivariate Analysis of Variance (MANOVA) with the factor group (DCM, TAAA) and the dependent variables white blood cell count (/µ), lactate (mmol/l), protein concentration (g/l), and Glucose (mg/dl). Note, that for this analysis only those participants for whom CSF routine samples could be taken were included (N = 23 DCM and N = 37 TAAA controls). Univariate post-hoc between subjects’ effects and partial eta squared (*ηp*^2^) effect sizes will be reported for the MANOVA results.

The parameters derived by ELISA were compared between groups using two Multivariate Analyses of Variance (MANOVAs) with the factor group (DCM, TAAA). The dependent variables of the angiogenic mediators Ang 2 (pg/ml), VEGF C (pg/ml), VEGF A (pg/ml) were considered in the first MANOVA, and, as inflammatory mediators, Rantes (pg/ml), IL 1 beta (pg/ml) and IL 8 (pg/ml) in the second MANOVA. Note that for the angiogenic mediators MANOVA the analysis consisted of N = 24 DCM patients and N = 23 TAAA controls for whom data was available. The sample for the inflammatory mediators MANOVA consisted of N = 21 DCM patients and N = 27 TAAA controls. Again, for all MANOVA results the univariate post-hoc between subjects’ effects and partial eta squared (*ηp*^2^) effect sizes will be reported.

### Ethical approval

All procedures performed in studies involving human participants were in accordance with the ethical standards of the institutional and/or national research committee [ocal ethics committee of the Medical Faculty of the XXX University (EK 164/13)] and with the 1964 Helsinki declaration and its later amendments or comparable ethical standards.

### Informed consent

Informed consent was obtained from all individual participants included in the study.

## Results

### Demographics and clinical findings

Patients of the DCM group (14 females; 14 males; *M*_age_ ± *SD*: 62.29 ± 10.82) and controls of the TAAA group (13 females; 25 males; *M*_age_ ± *SD*: 61.71 ± 15.04) had no differences in age and gender distribution of any statistical significance. However, the groups significantly differed according to clinical status [*M*_mJOA_ ± *SD*: DCM 10.08 ± 3.33 vs. TAAA 17.32 ± 1.23 (*p* < 0.001); *M*_NDI_: DCM 47.38 ± 19.71 vs. TAAA 5.32 ± 8.56 (*p* < *0.001*)]. Five Patients of the DCM group presented with mild clinical signs of myelopathy, 10 with moderate status and 13 patients with severe clinical symptoms concerning mJOA scoring (Table 1).

**Table 1:** Demographic and clinical findings of patients (DCM) and controls (TAAA).

Variables	Groups		Test statistic	Significance
	DCM *N*_total_ = 28	TAAA *N*_total_ = 38		
	M [SD]	M [SD]		p*
Age (years)	62.3 [10.8]	61.7 [15.0]	*t-test*	.69
mJOA Score	10.1 [3.3]	17.3 [1.2]	*t-test*	**< .001**
Neck disability Index	47.4 [19.71]	5.3 [8.56]	*t-test*	**< .001**

	Groups		Test statistic	Significance
	DCM *N*_total_ = 28	TAAA *N*_total_ = 38		
	N [%]	N [%]		p*
Females	14 [50.0]	13 [34.2]	*Chi*^2^	.14
Smokers	4 [14.2]	6 [15.8]		
mJOA symptoms				
Duration < 6 months	10 [35.7]			
Duration > 6 months	18 [64.3]			
Mild + moderate	15 [53.6]			
Severe	13 [46.4]			
MRI findings				
Positive TW2 signal	19 [67.9]			
Multisegmental	23 [82.1]			
Monosegmental	5 [17.9]			

*CSF* cerebrospinal fluid, *DCM* Degenerative cervical myelopathy, *M* mean, *mJOA* modified Japanese Orthopedic Association*, SD* standard deviation, *TAAA* thoracic abdominal aortic aneurysm.

Bold values indicate significance level of p < .05.

### Laboratory findings

#### CSF routine values

Standard routine values of CSF were recorded in 23 patients of the DCM group and 37 patients of the TAAA group. References for standard CSF values: Lactate (1.1–2.4 mmol/l), Protein (0.13–0.40 g/l), Glucose (40–70 mg/dl), White blood cell count (< 5/μl).

The MANOVA revealed significant group differences (F(55,4) = 14.1; *p* < 0.001; *ηp*^2^ = 0.51). Post hoc analyses showed significantly higher concentrations of protein in CSF in DCM patients compared to TAAA controls (*M*_*DCM*_ ± *SD*: 0.6 ± 0.2; *M*_*TAAA*_ ± *SD*: 0.3 ± 0.1; F(1,58) = 54.1, *p* < 0.001, *ηp*^2^ = 0.48). All other tests between the groups proved insignificant (all *p* > 0.05; Table 2). Table 2 displays the group means and standard deviations as well as the between groups effects.

**Table 2:** CSF routine values of patients (DCM) and controls (TAAA).

CSF routine values	DCM N = 23 M [SD]	TAAA N = 37 M [SD]	p*	*ηp*^2^
Lactate (mmol/l) (Ref: 1.1–2.4 mmol/l)	1.62 [0.3]	1.5 [0.4]	.17	.03
Protein (g/l) (Ref: 0.13–0.40 g/l)	0.6 [0.2]	0.3 [0.1]	**< .001**	**.48**
Glucose (mg/dl) (Ref 40–70 mg/dl)	58.9 [10.0]	58.7 [15.4]	.96	.00
White blood cell count (/μ) (Ref < 5 /μl)	1.8 [1.8]	1.5 [1.3]	.49	.01

*CSF* cerebrospinal fluid, *DCM* degenerative cervical myelopathy, *TAAA* thoracic-abdominal aortic aneurysm*, M* = mean*, SD* = standard deviation, *mmol/l* millimole per liter, *g/l* gram per liter, *mg/dl* milligram per decilitre.

*p values reflect the univariate post-hoc between group tests revealed by the significant MANOVA; *ηp*^2^ = partial eta squared effect size.

Bold values indicate significance level of p < .05.

#### Blood-spinal cord barrier

**Figure 2:**
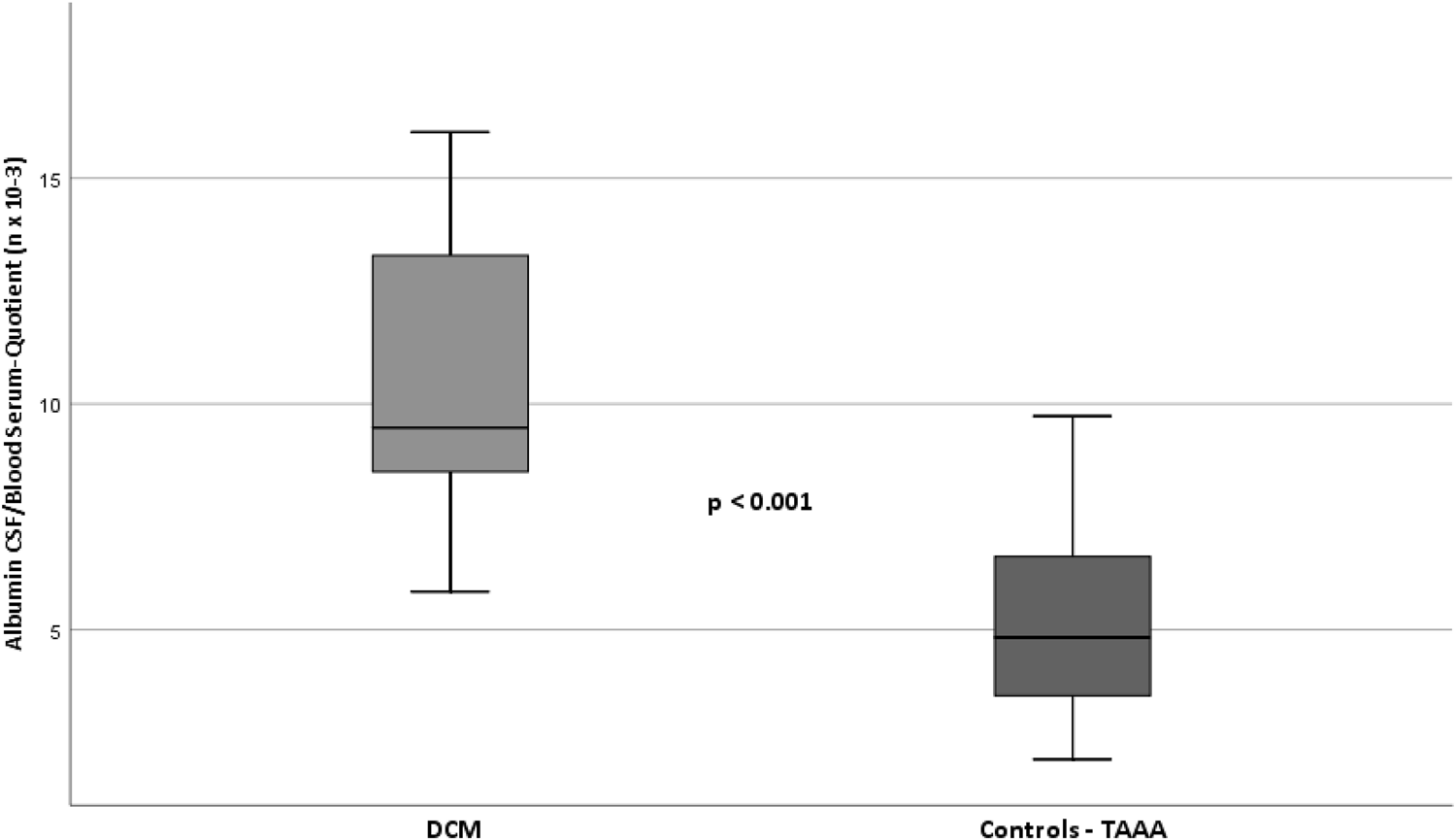
Significant differences of Albumin CSF/Blood Serum-quotients (AlbuminQ n × 10–3) between DCM and controls, as sign of blood spinal cord barrier disruption in DCM patients. *CSF* cerebrospinal fluid, *DCM* Degenerative cervical myelopathy, *TAAA* thoracic aortic abdominal aneurysm. Italic values indicate significance level of p < .05.

According to the Reibers´ diagnostic, patients of the myelopathy group presented a mild to moderate BSCB disruption (*M*_*DCM*_ ± *SD*: 10.5 ± 3.1; *M*_*TAAA*_ ± *SD*: 5.2 ± 1.9; *p* < 0.001). In contrast to the myelopathy group, participants of the control group did not show impairment of BSCB at all, as published by our research team recently^56^. See Fig. 2.

#### CSF ELISA examination

For the angiogenic mediators MANOVA the analysis consisted of N = 24 DCM patients and N = 23 controls for whom this specific data was extractable. The between groups MANOVA reached significance (F(3,42) = 4.5, *p* < 0.01, *ηp*^2^ = 0.3). Post-hoc univariate analyses revealed significant differences between groups for Ang 2 (*M*_*DCM*_ ± *SD*: 267.1 ± 81.9; *M*_*TAAA*_ ± *SD*: 408.6 ± 177.1; F(1,44) = 12.4, *p* < 0.01, *ηp*^2^ = 0.22). Moreover, for VEGF C, we found significant differences between groups (*M*_*DCM*_ ± *SD*: 152.2 ± 96.1; *M*_*TAAA*_ ± *SD*: 222.4 ± 140.3; F(1,44) = 4.4, *p* < 0.05, *ηp*^2^ = 0.09) while VEGF A did not reveal significant between group differences (*M*_*DCM*_ ± *SD*: 15.3 ± 8.5; *M*_*TAAA*_ ± *SD*: 15.4 ± 13.0; F(1,44) = 0.001, *p* = 0.98, *ηp*^2^ = 0.00).

**Figure 3:**
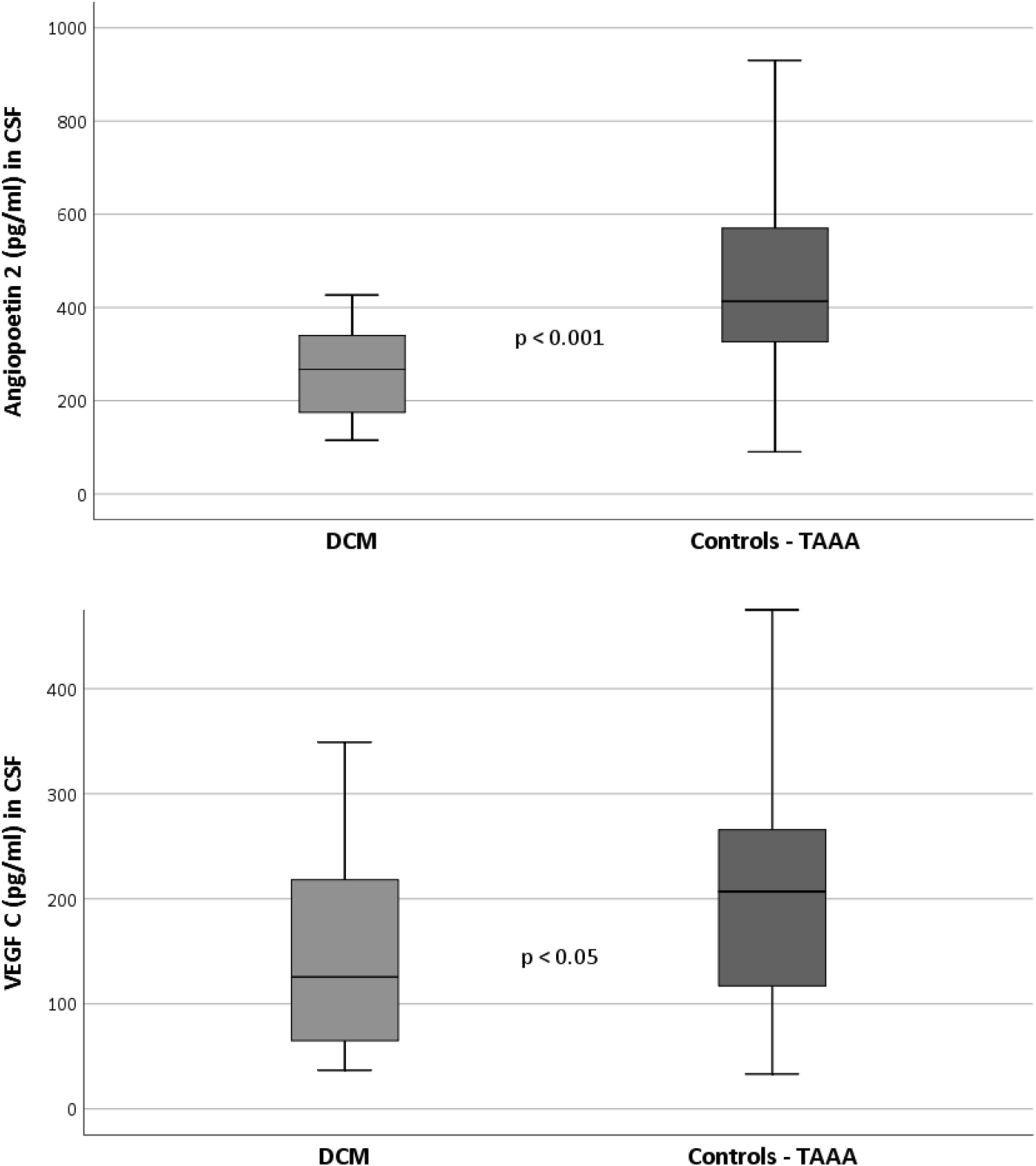
Significant differences of mediator concentrations (Angiopoietin 2 and VEGF C (pg/ml)) in CSF of DCM group and controls. *CSF* cerebrospinal fluid, *DCM* Degenerative cervical myelopathy, *pg/ml* picogram/milliliter. Italic values indicate significance level of p < .05.

The MANOVA including the inflammatory mediators consisted of N = 21 DCM patients and N = 27 controls for whom data samples were usable. The between groups MANOVA on the inflammatory mediators did not reach significance (F(3,43) = 0.95, *p* = 0.42, *ηp*^2^ = 0.06). Thus, all post-hoc univariate between group comparisons remained insignificant as well. Table 3 displays all variables’ means and standard deviations as well as the two MANOVAs’ post hoc univariate between group effects and effect sizes. The results of significant different mediator concentrations between the groups are displayed in Fig. 3 for Ang 2 and VEGF C.

**Table 3:** CSF ELISA testing of patients (DCM) and controls (TAAA).

CSF ELISA testing	DCM	TAAA	P*	*ηp*^2^
	M [SD]	M[SD]		
Angiogenic mediators	N = 24	N = 23		
Angiopoietin 2 (pg/ml)	267.1 [81.9]	408.6 [177.1]	**< .001**	***.22***
VEGF C (pg/ml)	152.2 [96.1]	222.4 [140.3]	***.04***	***.09***
VEGF A (pg/ml)	15.3 [8.5]	15.4 [13.0]	.98	.00
Inflammatory mediators	N = 21	N = 27		
IL1 beta (pg/ml)	1.7 [4.6]	1.2 [1.0]	.60	.01
IL 8 (pg/ml)	72.6 [74.8]	291.1 [721.8]	.14	.05
Rantes (pg/ml)	3.2 [10.0]	2.8 [3.3]	.88	.00

*CSF* cerebrospinal fluid, *DCM* degenerative cervical myelopathy, *pg/ml* picogram per milliliter, *M* mean*, SD* standard deviation, *TAAA* thoracic-abdominal aortic aneurysm.

*p values reflect the univariate post-hoc between group tests revealed by the MANOVAs; *ηp*^2^ = partial eta squared effect size.

Bold values indicate significance level of p < .05.

Between groups MANOVA of DCM patients with different duration of clinical signs of myelopathy (> < 6 months) on the inflammatory mediators (F(3,14) = 0.56; *p* = 0.65; *ηp*^2^ = 0.1) and angiogenic mediators revealed no significant difference. Table 4 further displays the between groups test (all not significant).

**Table 4:** CSF ELISA testing of patients (DCM) >  < 6 month.

CSF ELISA testing	DCM > 6 month	DCM < 6 month	P*	*ηp*^2^
	M [SD]	M [SD]		
Angiogenic mediators	N = 13	N = 9		
Angiopoietin 2 (pg/ml)	250.0 [85.3]	275.8 [76.3]	*.52*	*.02*
VEGF C (pg/ml)	162.0 [103.6]	149.2 [97.8]	*.69*	*.01*
VEGF A (pg/ml)	15.6 [9.8]	14.4 [7.5]	.73	.01
Inflammatory mediators	N = 11	N = 8		
IL1 beta (pg/ml)	.8 [.4]	3.2 [7.6]	.35	.06
IL 8 (pg/ml)	72.3 [95.8]	69.3 [50.5]	.90	.00
Rantes (pg/ml)	5.3 [13.7]	0.8 [.6]	.33	.06

*CSF* cerebrospinal fluid, *DCM* degenerative cervical myelopathy, *pg/ml* picogram per milliliter, *M* mean*, SD* standard deviation, *TAAA* thoracic-abdominal aortic aneurysm.

*p values reflect the univariate post-hoc between group tests revealed by the MANOVAs; *ηp*^2^ = partial eta squared effect size.

Italic values indicate significance level of p < .05.

## Discussion

Patients and controls had similar demographic characteristics, but clear and significant differences concerning clinical neurological status. Standard CSF values showed significant differences in higher protein (g/l) concentrations in the DCM group. These results are related to BSCB disruption of DCM patients in our study. CSF protein composition is mainly based on albumin^63^. According to Reibers’ diagnostic, higher albumin levels in CSF (combination of CSF/bloodserum quotient and age-related references) present the main characteristic of BSCB impairment^61,64,65^. In our study, DCM patients presented a BSCB disruption. This explains higher albumin values in CSF samples of DCM patients compared to controls. Additionally, the status of BSCB provides a better way to interpret the results between the two groups. The BSCB, which is not compromised in the control group, seems to be the main distinguishing factor in identifying a reliable control group to compare different alterations of mediators regarding inflammation and angiogenesis. Although controls were suffering from a vascular pathology, samples of CSF were taken from an immune privileged and enclosed intrathecal space. Thus, systemic reactions of other pathologies are of very limited influence due to the intact BSCB in TAAA patients^64–66^.

The concentrations of evaluated inflammatory factors in CSF neither revealed to be significant between patients and controls, nor between patients with shorter (< 6 months) and longer (> 6 months) clinical signs of myelopathy. IL1 beta and Rantes were in part under the detection limit or only present in very low concentrations in CSF in all groups. Similar observations of CSF inflammatory mediator concentrations were described previously by Nagashima et al.^3,67^. In a retrospective study, Ito et al. described a significantly higher concentration of IL8 in patients with DCM compared to a control group^3^. These findings are contrary to our findings of non-significant group differences with regard to the inflammatory mediators/IL-8 with lower CSF values of IL8 compared to our control group. However, there are some differences in study design between our study and Ito et al. including prospective versus retrospective study design, the numbers of control patients (38 TAA patients versus 12 Trauma patients), different ELISA Kits (ThermoFisher versus BD Biosciences), and different approaches in the clinical evaluation of the presence of an accompanying DCM in the control group (missing mJOA and NDI valuation in Ito et al.). These differences make it difficult to compare the studies’ findings. In addition, we are unable to exclude that the vascular pathology of our control group has no influence on the intrathecal cytokine values. Therefore, we conclude that IL-8, as inflammatory mediator, plays a secondary role in the pathogenesis of DCM in our cohort. In our study, pathomechanisms seem to be dominated by angiogenetic mediators. Our ongoing research consistently shows clearly that the BSCB in the control group is intact and that we can consider transfer from the serum to the CSF highly unlikely^56^. Nevertheless, proinflammatory cytokines are known to be up-regulated immediately in SCI^68^. Even in DCM, these immune-mediated responses of inflammation have been detected in the early and late onset of compression, associated with Fas-mediated apoptosis^5,8^. Our results and the results of comparable study groups indicate that inflammatory reactions and their mediators are restricted to the spinal cord parenchyma and are not detectable in the intrathecal space/CSF^18^.

However, the evaluation of angiogenic mediators revealed significant differences in concentrations in CSF between patients and controls. Ang 2 as well as VEGF C presented significantly reduced concentrations in CSF in DCM patients.

Angiogenesis is a complex process involved in the formation and interaction of multiple vascular regulating factors and expression of Ang 2 is up-regulated at sites of vascular remodeling following hypoxia^69–71^. In this context, Durham-Lee and colleagues observed a significant increase of Ang 2 expression after day 3 post trauma, persisting until day 56 after SCI^41^. Consistent with these findings, a constant downregulation of VEGF and transient decrease of Ang 1 have also been reported in acute spinal cord injury^50^. On the other hand, contrary to Durham-Lee and Ritz, but similar to our findings, Kim et al. described a decrease of Ang 2 using an experimental chronic hypoperfusion model^41,72^. In their study, Hippocampal Ang 2 expression also increased at 1 week, but after 4- and 8-weeks Ang 2 expression decreased. In our cohort, the DCM group had symptoms for a mean of 10 months. It is conceivable that Ang 2 could play a pathophysiological role in DCM in terms of hypoperfusion. The fact that we had that we had slightly higher Ang 2 values ​​in the group with a short anamnesis (< 6 months symptoms of DCM) compared to the group with a longer anamnesis (> 6 months of DCM) supports this assumption. However, these differences were not significant. VEGF C/VEGFR 3 may be involved in the glial reaction and possibly in the recruitment of monocytic macrophages during ischemic insults, contributing to inflammation in the ischemic brain^73–76^. Furthermore, VEGF-C affects macrophage migration via VEGFR 3, and VEGFR 3 modulates adaptive immunity by mediating chemotaxis of antigen-presenting (dendritic) cells^73,76,77^. Park et al. demonstrated a significant increase in the expression of VEGF C and its tyrosine kinase receptor, VEGFR 3, in the white matter of EAE rats, and in areas with a BSCB dysfunction. They observed a peak of VEGF C and VEGFR 3 expression after day 13–15 postimmunisation. Later, in the chronic phase (10 weeks), the values declined, but were still increased compared to controls. Expression of VEGFR 3 mRNA in the spinal cord was observed in gray matter neurons and in a subset of white matter astrocytes, but no resting ramified microglia. Their observations demonstrated that a population of round VEGFR 3–expressing cells appeared in the vicinity of blood vessels with the BBB impairment. Contrary to Park, in our entire DCM group we observed significantly degraded VEGF C values compared to our control group. Furthermore, we had lower, but not significant, VEGF C values in the group with a short anamnesis (< 6 months symptoms of DCM) compared to the group with a longer anamnesis (> 6 month of DCM). These differences between our findings and Park et al. are confusing at first, but the differences may be due to the disease model. DCM is more of a slowly progressing and chronic illness. Accordingly, a comparison with acute illnesses (e.g. SCI and EAE) is challenging, but due to the lack of more suitable alternatives, it is the only option, However, it is quite conceivable that the increased VEGF C expression in our DCM group with longer anamnesis (> 6 months) could be related to an increased inflammatory response and a longer-lasting disorder of the BSCB dysfunction triggered by VEGF C.

Furthermore, in an immunohistochemical study, Sano et al. described significantly higher VEGF C values in vessel tissue in 20 patients with abdominal aortic aneurysms compared to a control group, but not elevated VEGF A values^78^. In their study, no CSF or serum levels of VEGF were measured. On the other hand, Kaneko et al. also reported a VEGF A overexpression in the macrophages infiltrating the aortic abdominal aneurysm (AAA) wall^79^. Wolanska et al. investigated 24 patients undergoing an elective open AAA repair^80^. In Western-blot measurement they also observed an increased expression of VEGF C in the AAA wall, compared to a normal aorta. In our study, we observed significantly higher VEGF C concentrations in the CSF of our control group as well, while VEGF A levels were statistically not significant between DCM patients and the control group. This reveals a discrepancy between these mediators. We therefore see the need for further experiments and investigation in this regard.

Thus, we are well aware that our control group could exhibit increased levels of VEGF’s in the aorta too, or even in serum in certain circumstances. Nevertheless, as mentioned above, we could show that the BSCB in our control group is intact and that the transfer from the serum to the CSF can be regarded as very unlikely.

Chronic compression of the spinal cord is considered to be associated with a reduced vascular supply and seems to be one of the pathophysiological components of DCM^81^. Mechanical distortion harms intrinsic transverse vessels/terminal branches, ending in endothelial cell loss and dysfunction^82^. These endothelial cell dynamics are crucial for the integrity and permeability of the BSCB^83^. Reduced angiogenic activity and mediator concentrations in CSF could lead to increased endothelial dysregulation and vascular instability, maintaining BSCB disruption, which is known to be one of the central pathomechanisms in acute and chronic neurodegenerative diseases (e.g. amyotrophic lateral sclerosis, multiple sclerosis, spinal cord injury, et cetera)^84–87^. The presence of BSCB disruption in DCM patients of our cohort underlines the findings of downregulation of angiogenic mediators, causing and maintaining secondary harm to the spinal cord apart from mechanical damage. The disruption of the BSCB in patients with DCM has been shown in a study by our research team recently^56^. These pathomechanisms of reduced angiogenic mediators, possibly maintaining the BSCB disruption, could be a therapeutic target in the treatment procedures of DCM patients, in addition to surgical intervention. Ang and VEGF are known to be neuroprotective and neurotrophic factors and therefore possible tools for new therapies^88^.

Limitations of this study have to be mentioned. Participants of the control group presented with a clear vascular pathology. Angiogenic reactions are known to be part of TAAA. To our knowledge, examinations of these factors have only been performed from serum and/or tissue samples itself^89–92^. In our study, controls did not present BSCB impairment at all. Thus, it is very unlikely that systemic endogenous reactions have influence on intrathecal alterations of concentrations of mediators. From this point of view, the controls provide a neurologically healthy control group, suitable for the investigation of intrathecal pathological processes. Furthermore, in this study we could not provide data which correlate with the clinical findings of the DCM patients regarding duration of symptoms and clinical severity. This could be due to the fairly low number of DCM patients, especially in subgroup analyses.

## Conclusion

Patients with DCM presented significantly decreased concentrations of angiogenic mediators in CSF. Reduced angiogenic activity and associated endothelial dysfunction may be a crucial factor for BSCB disruption in DCM, causing ongoing secondary harm to the spinal cord.
